# Supplementary material for: Subducted carbon weakens the forearc mantle wedge in a warm subduction zone
Source: Nat Commun. 2024 Aug 26;15:7159. doi: 10.1038/s41467-024-51476-6 (PMC11347577; doi:10.1038/s41467-024-51476-6)
Supplement: Supplementary file 1 — Supplementary Information [file 41467_2024_51476_MOESM1_ESM.pdf]

Supplementary Information for

# Subducted carbon weakens the forearc mantle wedge in a warm subduction zone

Ryosuke Oyanagi & Atsushi Okamoto

## Supplementary Discussion

### 1. Mineral assemblage of subducting sediments and the nature of sediment-derived fluid

Supplementary Fig. 1a shows the predicted amount of water and carbon hosted in the metasedimentary rocks in relation to the geotherm along northeast Japan. Our prediction suggest dehydration occurs mainly at 0.7–0.9 GPa ($\Delta m_{\text{fluid}}$ = −0.19 wt%) and 2.0–2.3 GPa ($\Delta m_{\text{fluid}}$ = −1.8 wt%), which corresponds to the breakdown of stilpnomelane and lawsonite + amphibole, respectively (Supplementary Fig. 1a). The predicted carbon concentrations are largely unchanged from 0.7 to 2.2 GPa (0.80 wt%), but decrease to 0.29 wt% at 2.4 GPa (Supplementary Fig. 2a). In our prediction, the carbon is solely hosted by graphite (0.4 vol%) at 0.7–2.5 GPa, and no carbonates are formed (Supplementary Fig. 1a). The predicted logarithm of the oxygen fugacity relative to the fayalite–magnetite–quartz buffer (ΔFMQ) of the fluids increases from −3.3 at 25 km to +0.1 at 30 km, and then decreases to −1.3 at 68 km (Supplementary Fig. 1b). The predicted ΔFMQ values then increase to −0.5 at 74 km and decrease to −1.7 at 80 km (Supplementary Fig. 1b). The predicted molecular species in the fluid are dominated by $H_2O$, with a molar fraction ($X_{H2O}$) of 0.99–1.00 at depths from 25–90 km (Supplementary Fig. 1c). The predicted molar fraction of $CO_2$ ($X_{CO2}$) is $<10^{-5}$ at 25–70 km, but increases to 0.005 at 75–90 km (Supplementary Fig. 1c).

Predicted modal abundances of the mineral phases in the metasedimentary rocks along the Nankai geotherm[1] (Supplementary Fig. 1d) suggest they become dehydrated at 0.6–2.5 GPa (~1.6 wt%), mainly due to the breakdown of chlorite (Supplementary Fig. 1d). Significant decreases in rock $H_2O$ contents (0.6 wt%) are inferred to occur at 0.7–0.8 GPa

(Supplementary Fig. 1d). Our prediction shows carbonates (calcite or dolomite; 5.6 vol%) are dominant as compared with graphite (0.5 vol%) from 0.6 to 2.0 GPa, whereas only graphite (0.5 vol%) is present from 2.2 to 2.5 GPa. The predicted rock carbon contents remain largely unchanged from 1.1 wt% at 0.4 GPa to 0.9 wt% at 2.0 GPa (Supplementary Fig. 1d), and then decrease to 0.4 wt% at 2.0–2.2 GPa, indicative of the dissolution of significant amounts of carbon in the fluids. The predicted ΔFMQ values of the fluids decrease gradually from +0.5 (at 20 km) to −0.1 (at 73 km), and decrease abruptly to −0.9 (at 80 km depth; Supplementary Fig. 1e). $X_{H2O}$ varies from 0.88 to 1.00 with increasing depth from 25 to 90 km. $X_{CO2}$ increases with depth from 0.002 at 20 km to 0.1 at 70 km (Supplementary Fig. 1f).

## 2. Influence of *t/L* on model predictions

Our model prediction was affected by the parameter $t/L$ (Myr $m^{-1}$) in equation (4), where $t$ (Myr) is the timescale of fluid infiltration and $L$ (m) is the length or thickness of the forearc mantle basement normal to the slab dip. Here we compare the model predictions for $t/L$ = 0.1, 1, and 10 (Myr $m^{-1}$). We considered two types of model scenarios. The first type simulates an increasing amount of fluid entering the reacting serpentinite (herein, the "batch" calculation), which was used to obtain the results presented in the main manuscript (Fig. 2e–h). The second type simulates the fractionation of fluid at each step of fluid addition (herein, the "fractional" calculation), simulating the evolution of the bulk composition of the rock in a relatively open system. The batch and fractional calculations were performed by mode 0 and mode 1 calculations in the VERTEX subprogram in Perple_X, respectively. The calculations were performed along the slab-top geotherm of the northeastern Japan and Nankai subduction zones[1]. The reacting fluids were those fluids in equilibrium with the subducting sediments in the northeastern Japan (Fig. 2a) and Nankai (Fig. 2b) subduction zones.

In the northeastern Japan subduction zone, the results for the batch (Supplementary Fig. 2a, b) and fractional (Supplementary Fig. 2c, d) calculations in the case of $t/L$ = 0.1 Myr $m^{-1}$ show no significant difference from the reactant (i.e., serpentinite). At 74 km depth, the batch calculation (Supplementary Fig. 2a, b) with $t/L$ = 0.1 Myr $m^{-1}$ did not yield a result due to numerical instability, but the fractional calculation with $t/L$ = 0.1 Myr $m^{-1}$ yields a talc + magnesite assemblage (Supplementary Fig. 2c, d). The batch calculation with $t/L$ = 1 Myr $m^{-1}$ (Supplementary Fig. 2e and f) yields an identical to that in Fig. 2e and f, respectively, and the results are described in the main manuscript. The results are largely similar to the result of the fractional calculation with

$t/L$ = 1 Myr m$^{-1}$ (Supplementary Fig. 2g and h). The batch calculation with $t/L$ = 10 Myr m$^{-1}$ (Supplementary Fig. 2i, j) did not yield a result due to numerical instability. The fractional calculation with $t/L$ = 10 Myr m$^{-1}$ (Supplementary Fig. 2k, i) yields a talc + quartz assemblage at 68–80 km depth, with minor carbonate (Supplementary Fig. 2i).

For the Nankai subduction zone, the results of the batch calculation with $t/L$ = 0.1 Myr m$^{-1}$ show no significant difference from the reactant (i.e., serpentinite). A small amount of chlorite and amphibole occur at 55–60 km depth (Supplementary Fig. 3a). A small amount of carbonate (magnesite) was also predicted at 35–78 km depth (Supplementary Fig. 3b). The results of the fractional calculation with $t/L$ = 0.1 Myr m$^{-1}$ show similar trends to the batch calculation (Supplementary Fig. 3c, d). The batch calculation with $t/L$ = 1 Myr m$^{-1}$ (Supplementary Fig. 3e, f) yields the same results as in Fig. 2g, h, respectively. The results are described in the main manuscript. The fractional calculation with $t/L$ = 1 Myr m$^{-1}$ (Supplementary Fig. 3g, h) yields similar results to those of the batch calculation. The talc fraction is relatively low around the MWC and increases with depth. The proportions of talc and magnesite increase in a similar manner with increasing depth (Supplementary Fig. 3h). The batch calculation with $t/L$ = 10 Myr m$^{-1}$ (Supplementary Fig. 3i, j) did not yield a result due to numerical instability. The fractional calculation with $t/L$ = 10 Myr m$^{-1}$ shows that the talc fraction decreases and the quartz fraction increases with increasing depth (Supplementary Fig. 3k, l). The occurrence of talc + carbonate rock is predicted at 35–47 km depth, and carbonate (magnesite) + quartz rock at 54–74 km depth (Supplementary Fig. 3k, l).

Taking $t/L$ = 10 Myr m$^{-1}$, the batch calculations for the northeastern Japan (Supplementary Fig. 2i, j) and Nankai (Supplementary Fig. 3i, j) subduction zones did not yield a result, whereas the fractional calculations yielded results (Supplementary Figs. 2k, l and 3k, l). Overall, the batch and fractional calculations yield similar results for both subduction zones. Therefore, although the results provided in the main manuscript (Fig. 2e–h) were obtained from the batch calculations, we can use the result of fractional calculations for various $t/L$ values (0.1, 1, and 10) to discuss the mineral assemblage at a range of $t$ and $L$ values.

## 3. Model results regarding the infiltration of sediment-derived fluid into anhydrous forearc mantle

The model results regarding the interaction of fluids derived from subducted sediment and hydrous mantle wedge (serpentinite) are presented in the main manuscript. Similar

calculations were conducted for an anhydrous mantle wedge. The mantle wedge peridotite was modeled using the composition of depleted mid-ocean-ridge-basalt sourced mantle (DMM)[2] (Supplementary Table 1). The $Fe^{3+}/\Sigma Fe$ ratio was set to 0.035[3] and *t/L* was set to 1 (Myr $m^{-1}$).

The results for the northeastern Japan and Nankai subduction zones are largely similar to the results obtained when using a serpentinite composition (Fig. 2e–h). For northeastern Japan, talc + chlorite + magnesite is predicted at 70–72 km depth and chlorite at 75–90 km depth (Supplementary Fig. 4a, b). In the Nankai subduction zone, talc is not predicted to occur at 35–40 km depth around the MWC, but the proportion of talc increases from 5 vol% at 41 km depth to 59 vol% at 73 km (Supplementary Fig. 4c). Carbonate minerals (dolomite + magnesite; 6–29 vol%) occurs at 35–78 km depth (Supplementary Fig. 4d). In both subduction zones, larger amounts of chlorite are predicted compared with a serpentinite composition (Fig. 2e–h), because the DMM is pyroxene-rich.

## 4. Modeling the dehydration of subducting altered oceanic crust

Fluids released from subducting altered oceanic crust (AOC) would infiltrate the mantle wedge, along with the fluids from subducting sediment[4–6]. Inorganic carbon in AOC occurs mainly in the upper basaltic portion of the crust[7–10] (300 m thickness)[11,12]. We modelled the stable mineral assemblages along the slab-top geotherm of the northeast Japan and Nankai subduction zones using a typical carbonated AOC basalt[13] (Supplementary Table 1). We used a $H_2O–CO_2$ solvent for modeling AOC in both subduction zones. Solute $CO_2$ species ($CO_{2,aq}$) were excluded from the calculations. The solid-solution models used for the AOC calculation are listed in Supplementary Table 3. To calculate the fluid flux ($J$; kg fluid $m^{-2}$ $Myr^{-1}$) derived from dehydration of the AOC during subduction, a similar equation was used as that for calculating the fluid flux from sediment (equation 3 in the main text), rewritten for AOC as follows:

$$J = \rho_{\mathrm{AOC}} \frac{hv}{l} \Delta m_{\mathrm{fluid}}, \qquad \text{(Supplementary Equation 1)}$$

where $\rho_{AOC}$ (kg $m^{-3}$) is the density of the AOC, $h$ (m) is the thickness of the AOC, $v$ (m $Myr^{-1}$) is the subduction velocity, and $l$ (m) is the along-dip length of the slab. $\Delta m_{fluid}$ is the change in fluid mass concentration (kg fluid $kg^{-1}$ rock) over interval $l$. The $v$ values were set to 100,000 and 40,000 m $Myr^{-1}$ for northeastern Japan and Nankai, respectively[14]. The

*l* value was calculated from the depth and horizontal distance of the thermal structures in northeastern Japan and Nankai. We assumed that *h* and *v* are constant.

In northeast Japan, the $H_2O$ content (4.5 wt%) shows a minimal decrease from 25 to 70 km depth (Supplementary Fig. 5a), but a decrease from 4.5 wt% at 70 km to 0.4 wt% at 80 km depth due to the breakdown of lawsonite + pumpellyite. In contrast, in the Nankai subduction zone, decreases in $H_2O$ content are predicted at depths of ~30 and 58–90 km due to the breakdown of chlorite and chlorite + epidote, respectively (Supplementary Fig. 5b).

For northeast Japan, the predicted fluid flux during the subduction of AOC increases from $12.6 \times 10^3$ kg m$^{-2}$ Myr$^{-1}$ at 72 km depth to $767 \times 10^3$ kg m$^{-2}$ Myr$^{-1}$ at 74 km depth, and then decreases to ~$3.0 \times 10^3$ kg m$^{-2}$ Myr$^{-1}$ at 90 km depth (Supplementary Fig. 5c). The predicted total flux (i.e., the sum of fluid flux from sediment and AOC) is zero at 32–68 km depth (Supplementary Fig. 5c). The total flux at 68–72 km depth is dominated by the flux from sediment and ranges from $2.6 \times 10^3$ to $44.8 \times 10^3$ kg m$^{-2}$ Myr$^{-1}$ (Supplementary Fig. 5c). The total flux at 72–90 km depth is dominated by fluid from AOC, and ranges from $4.0 \times 10^3$ to $1050 \times 10^3$ kg m$^{-2}$ Myr$^{-1}$. Below the depth of the island arc Moho in the Nankai subduction zone (35 km depth), dehydration of AOC is predicted at 58–90 km depth (Supplementary Fig. 5d). The predicted fluid flux during the subduction of AOC increases from $9.0 \times 10^3$ kg m$^{-2}$ Myr$^{-1}$ at 58 km depth to $58.4 \times 10^3$ kg m$^{-2}$ Myr$^{-1}$ at 67 km depth, and then decreases gradually to $2.0 \times 10^3$ kg m$^{-2}$ Myr$^{-1}$ at 82 km depth (Supplementary Fig. 5d). At 36–56 km depth, the predicted total fluid flux (1.5–$7.3 \times 10^3$ kg m$^{-2}$ Myr$^{-1}$) is dominated by fluid from sediment, whereas fluid from AOC is dominant at depths of 56–90 km (2.0–$58.4 \times 10^3$ kg m$^{-2}$ Myr$^{-1}$; Supplementary Fig. 5c).

The total flux was used to constrain the mineral assemblage in the northeastern Japan and Nankai subduction zones, with *t/L* set to 1 (Myr m$^{-1}$). In northeastern Japan, serpentine and chlorite are predicted to occur at depths of 68 and 77–90 km, respectively (Supplementary Fig. 5e), without carbonate minerals (Supplementary Fig. 5f). Compared with the model results that consider only sediment dehydration (Fig. 2e, f), the total flux shows greater amounts of talc and chlorite at depths of >70 km. In the Nankai subduction zone, the model calculation suggests that the proportion of talc increases with depth (Supplementary Fig. 5g), as also observed for the model results considering the dehydration of sediment (Fig. 2g, f). The proportion of talc increases from 0.6–5.0 vol% around the MWC (35–40 km depth) to 56 vol% at 60 km depth (Supplementary Fig. 5g). Compared with the results obtained when considering only sediment dehydration (Fig.

2g), an increase in the proportion of talc is predicted at depths of 58–63 km (Supplementary Fig. 5g) as a result of fluid flux from AOC (Supplementary Fig. 5d, g). At 68 km depth, where the total flux shows a peak, magnesite + quartz occur without hydrous minerals (Supplementary Fig. 5h). The results indicate magnesite occurrence without hydrous minerals at depths of 82–90 km.

In the case that fluid fluxes from AOC are considered, enhanced fluid fluxes are predicted at depths of >72 km and >58 km in the northeastern Japan (Supplementary Fig. 5c) and Nankai (Supplementary Fig. 5d) subduction zones. The predicted depth of AOC dehydration is largely consistent with previous studies[6]. Because dehydration of AOC does not occur around the MWC in the Nankai subduction zone, we conclude that the coupled dehydration of subducting sediment and AOC may enhance the contrast between a talc-poor region around the MWC and a talc-rich region at greater depths.

## 5. Effect of underplating of subducting sediments

In the model, we assumed that the thickness of subducted sediment ($h$) remains similar with depth, although sediment underplating may reduce the flux of sediment delivery to the deep Earth. The thickness of subducting sediment at forearc to subarc depths is geophysically unobservable, but on-land exposures of paleo-subduction zones suggest that underplated sediment makes up 20%–85% of the total subducted sediment[15–17]. With increasing proportion of underplated sediment, the fluid flux into the overlying forearc mantle would originate mainly from AOC. In the Nankai subduction zone, the calculation with AOC shows that the fluid flux around the MWC (35 km) and at greater depths (>58 km) is mainly from subducting sediment and AOC, respectively (Supplementary Fig. 5d). Therefore, with increasing proportion of underplated sediment, the fluid flux around the MWC may decrease, while that at greater depths may show little change. These depth variations in fluid flux related to sediment underplating would enhance the heterogeneity in talc distribution. Consequently, sediment underplating also results in a heterogeneous distribution of talc, and subduction megathrusts in warm subduction zones are expected to show down-dip variations in talc occurrence.

## 6. Calculations with and without carbon

Based on calculations of the infiltration of sediment-derived fluid into the mantle rocks under various $P$–$T$ conditions along the slab top in Nankai and northeastern Japan, we

estimated the amount of fluid required for the appearance of talc ($\xi_{Tlc}$) (Supplementary Fig. 6). In the northeastern Japan subduction zone, $\xi_{Tlc}$ first increases from 360 mol kg$^{-1}$ at 0.6 GPa to 1000 mol kg$^{-1}$ at 0.7 GPa, and then decreases to 106 mol kg$^{-1}$ at 2.0 GPa in the carbon-bearing system. Even if carbon is excluded from the system, $\xi_{Tlc}$ does not change significantly, ranging from 30 to 400 mol kg$^{-1}$ at pressures of 0.6 to 2.5 GPa. In the Nankai subduction zone, $\xi_{Tlc}$ decreases with increasing $P$ and $T$, from $\xi$ = 166 mol kg$^{-1}$ at 0.6 GPa to 9 mol kg$^{-1}$ at 2.1 GPa (Supplementary Fig. 6). For comparison, similar calculations were conducted with carbon-free sediments, resulting in $\xi_{Tlc}$ = 327 mol kg$^{-1}$ at 0.6 GPa to $\xi_{Tlc}$ = 41 mol kg$^{-1}$ at 2.1 GPa (Supplementary Fig. 6). The obtained $\xi_{Tlc}$ values are two to four times higher than in the carbon-bearing case.

## 7. Parameter studies on subducted carbon in sediment

The key variables in the present study are the subduction geotherm (i.e., $P$–$T$ conditions), whole-rock sediment composition, total carbon (TC) contents, and fraction of organic carbon relative to TC ($F_{OC}$). These parameters vary in each subduction zone. We conducted thermodynamic calculations using various parameters to understand the sensitivities of the parameters to metasomatism of the mantle wedge. The calculation settings are summarized in Supplementary Table 4. The calculations used the global average of subducting sediments (GLOSS)[18] as the whole-rock composition of the sediments. GLOSS contains 3.01 wt% $CO_2$, which corresponds to ~8200 mg kg$^{-1}$ TC. Because the $F_{OC}$ of GLOSS is unknown, we conducted thermodynamic calculations using GLOSS for selected $F_{OC}$ values (0.1, 0.5, and 0.9) at the geotherm of Nankai and northeastern Japan (calculations 1–6 in Supplementary Table 4). Moreover, we conducted thermodynamic calculations using GLOSS with modified TC values: GLOSS with 4100 mg kg$^{-1}$ TC (calculations 7–12 in Supplementary Table 4) and 16,200 mg kg$^{-1}$ TC (calculations 13–18 in Supplementary Table 4) for selected $F_{OC}$ values (0.1, 0.5, and 0.9). These calculations constrain the effects of the subduction geotherm, TC, and $F_{OC}$. The bulk rock composition used for each calculations were listed in the Supplementary Table 5.

Concentrations of carbon in the fluids ($m_C$) equilibrated with the sediments are shown in Supplementary Fig. 7a. Despite the changes in TC and $F_{OC}$, the concentrations of carbon are broadly similar for each subduction zone. For example, in the Nankai subduction zone, the $m_C$ values for TC = 4100, 8200, and 16400 ppm, and 0.6–2.2 GPa are 0.5–4.1, 0.5–6.1, and 0.5–7.4 mol kg$^{-1}$, respectively. In the northeastern Japan subduction

zone, the $m_C$ values for TC = 4100, 8200, and 16400 ppm, and 0.6–2.2 GPa are 0.1–0.5, 0.1–0.6, and 0.2–0.9 mol $kg^{-1}$, respectively. In both subduction zones, $F_{OC}$ = 0.1 leads to relatively high $m_C$ values as compared with $F_{OC}$ = 0.5 and 0.9 (Supplementary Fig. 7a–c). In all calculations, $m_C$ is high in the Nankai subduction zone (warm subduction zone) as compared with the northeastern Japan subduction zone (cold subduction zone), suggesting the subduction geotherm controls $m_C$ in metamorphic fluids.

Concentrations of Si in the fluids ($m_{Si}$) equilibrated with the sediments are shown in Fig. S7d–f. In the Nankai subduction zone, less variation of $m_{Si}$ values with TC and $F_{OC}$ are observed, ranging from 0.07 to 1.0 mol $kg^{-1}$ at 0.6–2.2 GPa, $F_{OC}$ = 0.1–0.9, and TC = 4100–16,400 ppm (Supplementary Fig. 7d–f). In the northeastern Japan subduction zone, $m_{Si}$ values vary from 0.03 to 0.15 mol $kg^{-1}$ at 0.6–2.2 GPa, $F_{OC}$ = 0.1–0.9, and TC = 4100–16,400 ppm. At 8200 and 16,400 ppm TC, $m_{Si}$ values at $F_{OC}$ = 0.1 are lower than those at $F_{OC}$ = 0.5 and 0.9 (Supplementary Fig. 7e–f). In general, $m_{Si}$ values are high in the Nankai subduction zone (warm subduction zone) as compared with the northeastern Japan subduction zone (cold subduction zone).

Supplementary Fig. 7g–i shows a summary of the calculations of infiltration of sediment-derived fluids into the mantle rocks under various *P*–*T* conditions along the slab-top of the Nankai and northeastern Japan subduction zones. In northeastern Japan, the $\xi_{Tlc}$ values for various $F_{OC}$ values are 60–300, 45–250, and 35–189 mol $kg^{-1}$ for sediments for 0.41, 0.82, and 1.62 wt% TC, respectively. In contrast, in the Nankai subduction zone, the $\xi_{Tlc}$ values for various $F_{OC}$ values are 10–182, 8–171, and 8–200 mol $kg^{-1}$ for sediments for 0.41, 0.82, and 1.62 wt% TC, respectively. The $\xi_{Tlc}$ values are typically low for the Nankai subduction zone as compared with those for the northeastern Japan subduction zone, suggesting the geotherm is the primary control on the efficiency of talc formation. Furthermore, the $\xi_{Tlc}$ values become smaller with increasing TC in the sediments in northeastern Japan, whereas this trend is less clear for the Nankai subduction zone. Therefore, increasing TC in the subducting sediments requires less fluid for talc formation in a cold subduction zone.

Even at the same geotherm, a low $F_{OC}$ value likely decreases the $\xi_{Tlc}$ value. For example, in the Nankai subduction zone, or for a calculation with sediments with 0.41 wt% TC (Supplementary Fig. 7g), the $\xi_{Tlc}$ values for $F_{OC}$ = 0.5 and 0.9 are similar, but the $\xi_{Tlc}$ values for $F_{OC}$ = 0.1 are lower, especially at lower *P* (<1.0 GPa). This trend is also observed when TC is increased (Supplementary Fig. 7h–i) in the northeastern Japan

subduction zone (Supplementary Fig. 7g–i). Therefore, low $F_{OC}$ (i.e., carbonate-dominated sediments) may enhance talc formation in the mantle wedge.

## 8. Effect of sediment type on talc formation in the forearc mantle

We conducted parameter studies to investigate the effect of sediment type on talc formation in the forearc mantle. We considered five types of sediment: carbonate sediment, chert, pelagic clay, terrigenous sediment, and turbidite.

The chemical compositions of the sediments are listed in Supplementary Table 6. The bulk rock composition of the carbonate sediment was taken from site 495 in Guatemala, from Plank and Langmuir[18]. We assumed that all carbon in the carbonate sediment was present as carbonate minerals ($F_{OC}$ = 0.00). For the other sediment types (chert, pelagic clay, terrigenous sediment, and turbidite), we combined the concentrations of organic and inorganic carbon reported by Clift[14] with the bulk compositions of Plank[19]. For chert and pelagic clay, we used the bulk rock compositions from site 801 at the Mariana Islands[18], along with organic and inorganic carbon concentrations from this site[14]. For terrigenous sediment, we used the bulk rock composition from the Antilles[18], and organic and inorganic carbon concentrations from the "Lesser Antilles mean" from Clift[14]. For turbidite, we used the bulk rock composition from site 178 in Alaska[18] along with organic and inorganic carbon concentrations from the same site[14]. The $Fe^{3+}/\Sigma Fe$ ratio was set to 0.23, based on the global average for metapelites[20]. The calculations were conducted along the slab-top geotherm of the northeastern Japan and Nankai subduction zones[1].

For northeastern Japan, the calculated fluids in equilibrium with the five types of sediment yield higher values of $m_C$ (0.1–2.0 mol $kg^{-1}$) than $m_{Si}$ (0.004–0.1 mol $kg^{-1}$; Supplementary Fig. 8a). The chert has the highest $m_C$ values (~2.0 mol $kg^{-1}$), whereas the carbonate sediment has the lowest (0.1–0.2 mol $kg^{-1}$). For the Nankai subduction zone, $m_C$ values (0.5–4.8 mol $kg^{-1}$) are an order of magnitude higher than $m_{Si}$ values (0.06–0.6 mol $kg^{-1}$; Supplementary Fig. 8b). The chert has the highest $m_C$ values (2.0–4.8 mol $kg^{-1}$), followed by pelagic clay (0.9–4.8 mol $kg^{-1}$). The $m_{Si}$ values are similar for the five types of sediment (Supplementary Fig. 8b).

For northeastern Japan, the calculated $H_2O$ content (Supplementary Fig. 8c) is high for pelagic clay (3.4–3.7 wt%), terrigenous sediment (3.9–4.5 wt%), and turbidite (3.5–4.5 wt%), and low for carbonate sediment (0.21–0.66 wt%) and chert (0.48 wt%). The chert shows minimal changes in $H_2O$ content with pressure. These trends are similar to those for the Nankai subduction zone (Supplementary Fig. 8d). The calculated $H_2O$

content is high for pelagic clay (1.8–2.2 wt%), terrigenous sediment (2.4–3.8 wt%), and turbidite (1.4–3.2 wt%), and low for carbonate sediment (0.11–0.23 wt%) and chert (0.31–0.51 wt%).

Using the calculated fluid compositions, we calculated the infiltration of sediment-derived fluid into the mantle rocks beneath the slab-top at the northeastern Japan and Nankai subduction zones. For northeastern Japan, the calculated $\xi_{Tlc}$ values (Supplementary Fig. 8e) increase in the order of chert, pelagic clay, turbidite, terrigenous sediment, and carbonate sediment. The chert shows the lowest $\xi_{Tlc}$ value (~20 mol kg$^{-1}$), with the other sediment types having $\xi_{Tlc}$ values of >100 mol kg$^{-1}$. The trends are similar for the Nankai subduction zone (Supplementary Fig. 8f), where the calculated $\xi_{Tlc}$ values increase in the order of chert (8–30 mol kg$^{-1}$), pelagic clay, carbonate sediment, turbidite, and terrigenous sediment.

The calculations indicate the effect of subducting sediment on talc formation in the forearc mantle. In both subduction zones, carbonate sediment and chert have low $\xi_{Tlc}$ values (Supplementary Fig. 8e, f). However, fluid flux might also control the formation of talc in the forearc mantle. The calculations considered only the latter effect. The carbonate sediment and chert in both subduction zones have low $H_2O$ contents and show minimal $H_2O$ loss with increasing pressure (i.e., with increasing depth; Supplementary Fig. 8c, d). Therefore, fluid flux from carbonate sediment and chert would be limited, as previously suggested[21], and subduction of these sediments might not result in significant talc formation in the forearc mantle.

In the northeastern Japan and Nankai subduction zones, the calculated $H_2O$ contents of the pelagic clay, terrigenous sediment, and turbidite show a decrease with increasing pressure (i.e., depth; Supplementary Fig. 8c, d), suggesting these types of sediment may be dehydrated during subduction. The calculated $\xi_{Tlc}$ values for these sediments are larger in northeastern Japan than at Nankai (Supplementary Fig. 8e, f), suggesting that the geotherm is the primary control on talc formation, as suggested from the results presented in Supplementary Discussion 7. Moreover, in both subduction zones, $\xi_{Tlc}$ increases in the order of pelagic clay, turbidite, and terrigenous sediment (Supplementary Fig. 8e, f). Therefore, the generation of even a small amount of fluid from subducting pelagic sediment may be sufficient for talc formation in the forearc mantle.

## 9. Prediction of the mineral assemblage in forearc mantle of the Cascadia subduction zone

We applied our thermodynamic approach to the Cascadia subduction zone. The chemical compositions of subducting sediment are provided in Supplementary Table 1. For the modeling of subducting sediment, we used the Cascadia sediment composition of Plank[19], although carbon contents were not provided. We used the organic and inorganic carbon contents of Cascadia sediment reported by Clift[14] in addition to the bulk compositions from Plank[19]. The $Fe^{3+}/\Sigma Fe$ ratio was set to 0.23, based on the global average for metapelites[20]. The calculations were conducted along the slab-top geotherm of the Cascadia subduction zone. The subduction velocity ($v$) was set to 42,000 m $Myr^{-1}$ [14] and we used the thickness of subducting sediments ($h$ = 400 m) from ref.[18].

Fluids in equilibrium with subducting sediment in the Cascadia subduction zone are carbon-rich, with C concentrations increasing from 1.9 mol $kg^{-1}$ at 25 km depth to 11.6 mol $kg^{-1}$ at 90 km (Supplementary Fig. 9a). The Si concentrations are an order of magnitude lower than the C concentrations, increasing from 0.1 mol $kg^{-1}$ at 25 km depth to 4.5 mol $kg^{-1}$ at 90 km. The mineral assemblage of the subducting sediment (Supplementary Fig. 9b) indicates that the amount of $H_2O$ in the rock decreases from 1.6 to 1.5 wt% at 31 to 34 km depth, and from 1.7 to 1.2 wt% at 41 to 73 km depth. These changes respectively correspond to the breakdown of chlorite + epidote + biotite and amphibole + mica. The calculated fluid flux from subducting sediment is 0.5–9.1 × $10^3$ kg $m^{-2}$ $Myr^{-1}$ from 31 to 34 km depth (Supplementary Fig. 9c). The fluid flux at depths below the island arc Moho (i.e., 35 km) increases from 0.7 × $10^3$ kg $m^{-2}$ $Myr^{-1}$ at 43 km depth to 13.9 × $10^3$ kg $m^{-2}$ $Myr^{-1}$ at 58 km depth, and subsequently decreases to 1.6 × $10^3$ kg $m^{-2}$ $Myr^{-1}$ at 73 km depth.

We now consider the predicted mineral assemblage in the forearc mantle of the Cascadia subduction zone for $t/L$ =1. The talc fraction increases from 2 to 60 vol% at 42–52 km depth (Supplementary Fig. 9d), and carbonate minerals (magnesite + dolomite) increase from 5 to 52 vol% (Supplementary Fig. 9e). The mineral assemblage at 54–65 km depth was not calculated due to numerical instability. Talc + serpentinite + carbonate (magnesite) is predicted to occur at depths of 67–69 km, and carbonate minerals are predicted to occur at 73 km depth without hydrous minerals (Supplementary Fig. 9d, e).

Overall, our model predicts a talc + carbonate assemblage in the Cascadia and Nankai subduction zones (Fig. 2g, h). In the former, the proportions of talc and magnesite show similar increases with depth (Supplementary Fig. 9d, e), suggesting these minerals are formed mainly by the infiltration of carbon-rich fluid (reaction 2) rather than silica-rich fluid (reaction 1).

## 10. Changes in seismic properties in the response to infiltration of metasediment-derived fluid into serpentinites

The P- and S-wave velocities for zero-porosity mineral aggregates were calculated using the program MinVel[22], which is a customized code based on ref[23]. The P- and S-wave velocities were calculated by excluding the hematite that was observed in our thermodynamic calculations because the MinVel dataset does not include hematite.

The thermodynamic model results were used to predict seismic velocities from the mineral proportions. For example, as the mantle wedge reacts with fluid originating from subducting sediments, the mantle wedge serpentinite is gradually modified to a rock consisting mainly of talc + carbonate (magnesite; Supplementary Fig. 10a). The serpentinite has $V_P$ = 6.7–7.0 km $s^{-1}$ and $V_S$ = 3.8–3.9 km $s^{-1}$, whereas the talc + magnesite rock has $V_P$ = 7.0 km $s^{-1}$ and $V_S$ = 4.2 km $s^{-1}$ (Supplementary Fig. 10b), suggesting that the changes in mineral assemblages in response to fluid infiltration would not result in significant changes in $V_P$ and $V_S$. Moreover, $V_P$ overlaps values for anhydrous (8.0–8.4 km $s^{-1}$) to fully hydrous mantle (6.6 km $s^{-1}$; Supplementary Fig. 10b)[6]. Therefore, distinguishing serpentinite and talc–carbonate rock solely based on seismic observations would be difficult, as previously suggested[24], and the low-velocity layer in the Nankai subduction zone could also be due to talc–carbonate layers at the bottom of the mantle wedge.

## Supplementary Figures

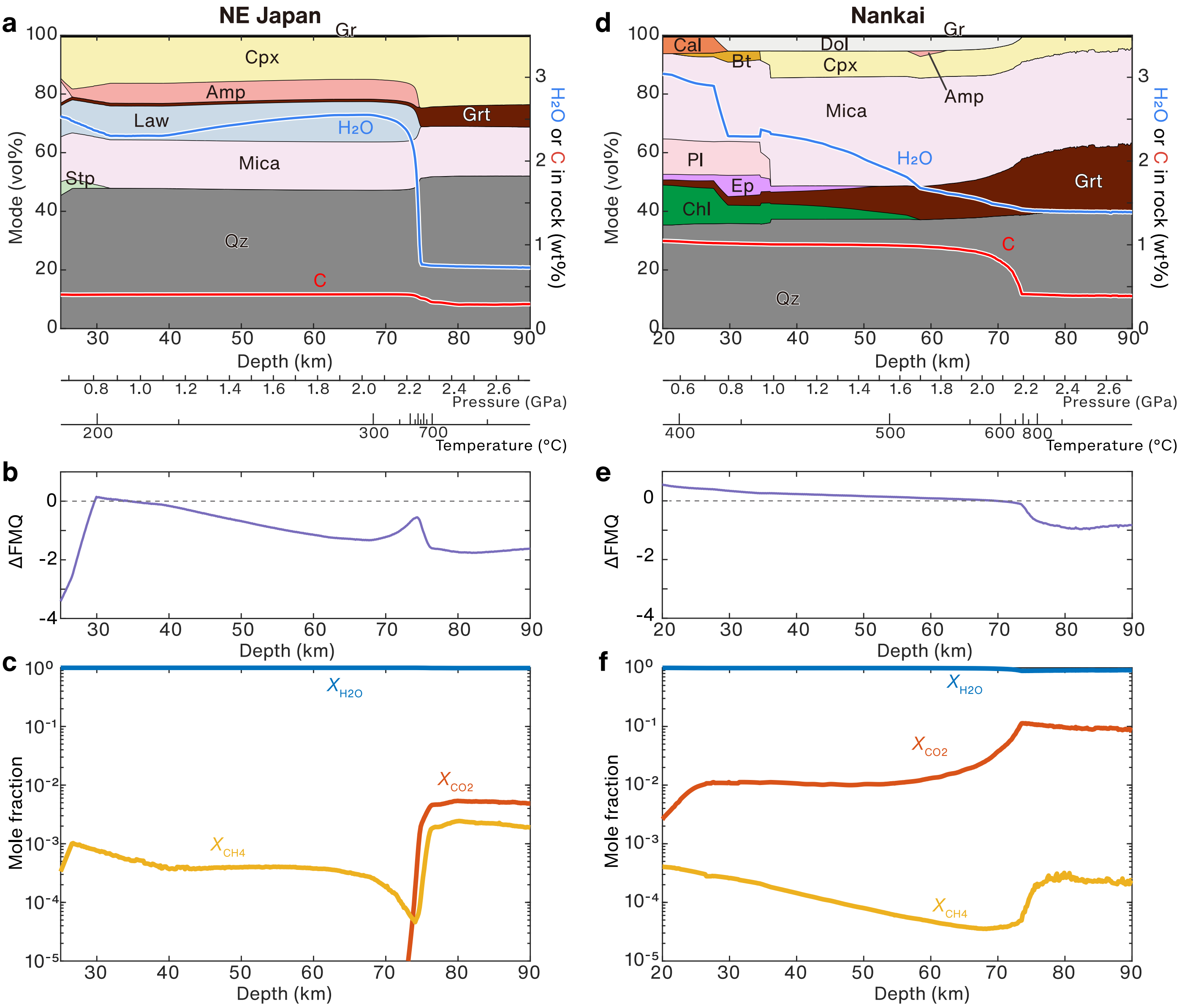


**Supplementary Figure 1.** | **Model predictions of the subducting sediments. a-c** Calculations along the *P*–*T* path of the northeastern Japan subduction zone. Predicted mineral phases with (**a**) $H_2O$ and C concentrations in the metasedimentary rocks, (**b**) oxygen fugacity, and (**c**) molecular species in the fluid. **d–f** Calculations along the *P*–*T* path of the Nankai subduction zone. Predicted mineral phases with (**d**) $H_2O$ and C concentrations in the metasedimentary rocks, (**e**) oxygen fugacity, and (**f**) molecular species in the fluid. Mineral abbreviations are after Whitney and Evans[25]: Qz = quartz, Stp = stilpnomelane, Law = lawsonite, Amp = amphibole, Cpx = clinopyroxene, Gr = graphite, Chl = chlorite, Ep = epidote, Pl = plagioclase, Bt = biotite, Grt = garnet, Cal = calcite, and Dol = dolomite.

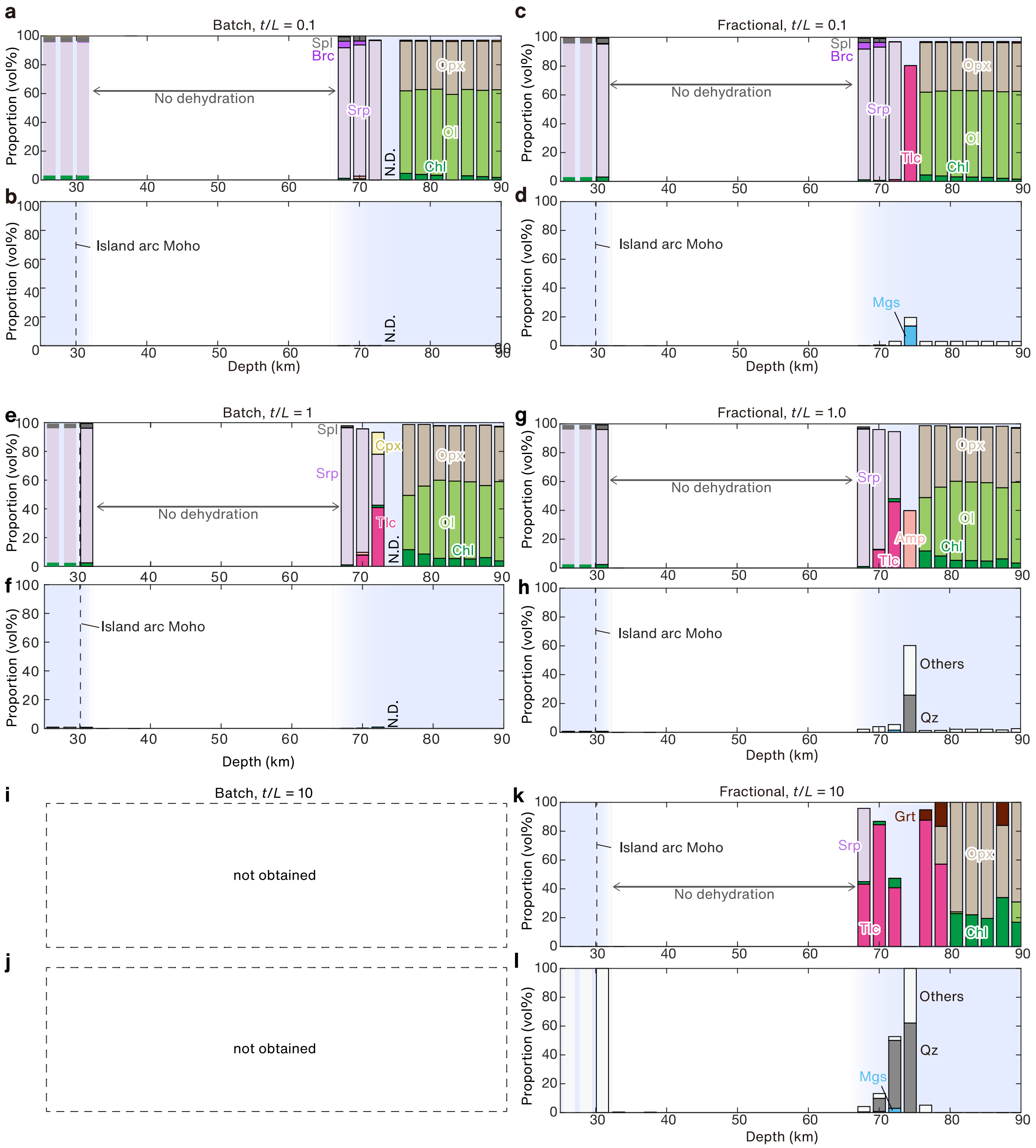


**Supplementary Figure 2.** | **Model predictions for the northeastern Japan subduction zone. a–b** Mineral proportions (**a**) and carbonate proportion (**b**) from the batch calculation with *t/L* = 0.1 (Myr $m^{-1}$). **c–d** Mineral proportions (**c**) and carbonate proportion (**d**) from the fractional calculation with *t/L* = 0.1 (Myr $m^{-1}$). **e–f** Mineral proportions (**e**) and carbonate proportion (**f**) from the batch calculation with *t/L* = 1 (Myr $m^{-1}$). These results are identical to those in Fig. 2**e** and **f**. **g–h** Mineral proportions (**g**) and proportions of carbonate, quartz, and other minerals (**h**) from the fractional calculation with *t/L* = 1 (Myr $m^{-1}$). **i–j** Mineral proportions (**e**) and carbonate proportion (**f**) were not obtained from the batch calculation with *t/L* = 10 (Myr $m^{-1}$). **k–l** Mineral proportions (**k**) and proportions of carbonate, quartz, and other minerals (**l**) from the fractional calculation with *t/L* = 10 (Myr $m^{-1}$). Mineral proportions in regions shallower than the island arc Moho are shown in light colors for reference. N.D. (not determined) indicates the mineral proportions were not calculated due to numerical instability. The blue and white backgrounds indicate the depths at which fluid supply into the mantle wedge was predicted and not predicted, respectively. Srp = serpentine; Tlc = talc; Chl = chlorite; Mgs = magnesite; Dol = dolomite; Qz = quartz.

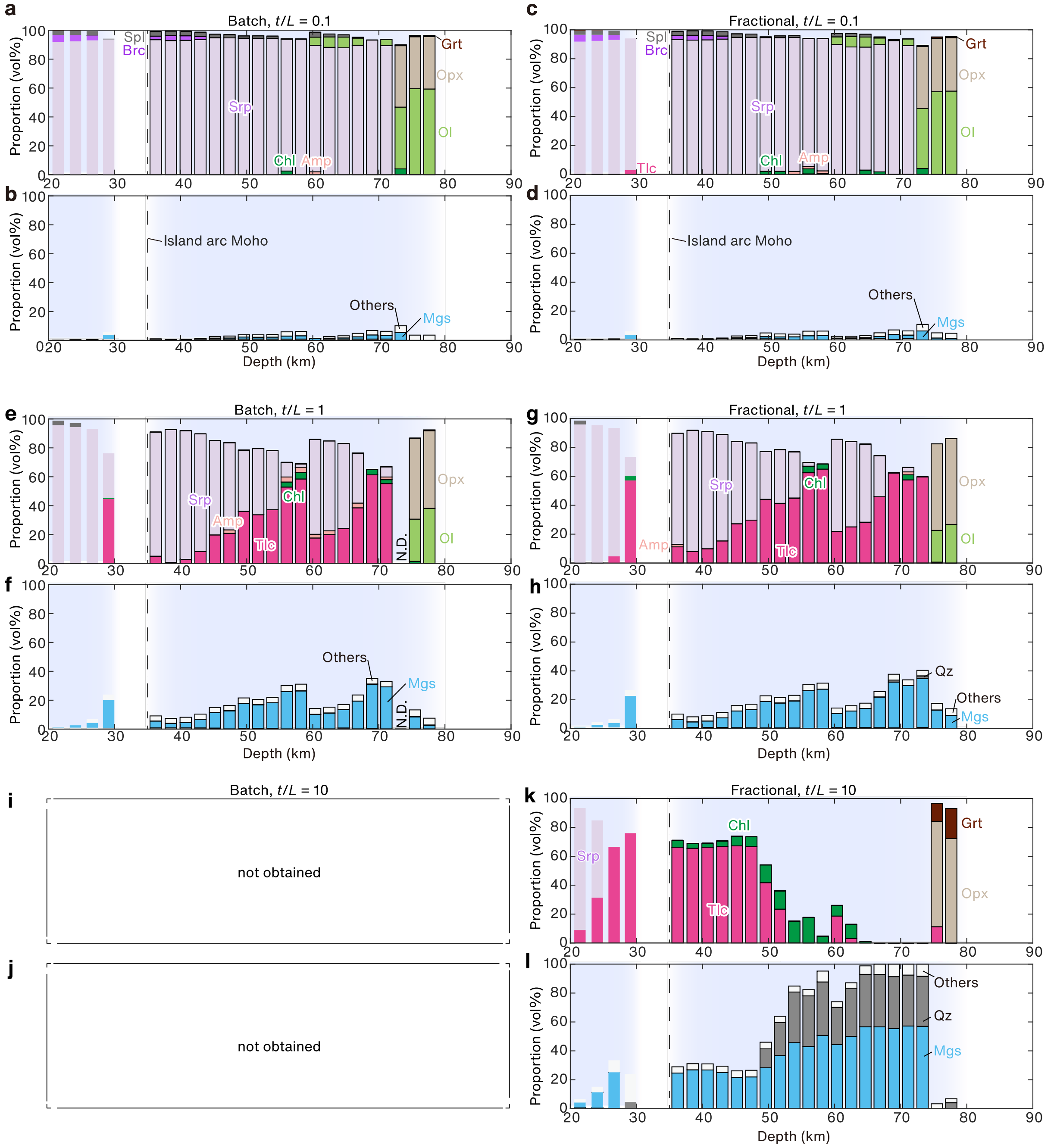


**Supplementary Figure 3.** | **Model predictions for the Nankai subduction zone. a–b** Mineral proportions (**a**) and carbonate (**b**) from the batch calculation with $t/L$ = 0.1 (Myr m$^{-1}$). **c–d** Mineral proportions (**c**) and carbonate (**d**) from the fractional calculation with $t/L$ = 0.1 (Myr m$^{-1}$). **e–f** Mineral proportions (**e**) and carbonate (**f**) from the batch calculation with $t/L$ = 1 (Myr m$^{-1}$). The results are identical to those in Fig. 2**e** and **f**. **g–h** Mineral proportions (**g**) and proportions of carbonate, quartz, and other minerals (**h**) from the fractional calculation with $t/L$ = 1 (Myr m$^{-1}$). **i–j** Mineral proportions (**e**) and carbonate (**f**) were not obtained from the batch calculation with $t/L$ = 10 (Myr m$^{-1}$). **k–l** Mineral proportions (**k**) and proportions of carbonate, quartz, and other minerals (**l**) from the fractional calculation with $t/L$ = 10 (Myr m$^{-1}$). Mineral proportions in regions shallower than the island arc Moho are shown in light colors for reference. N.D. (not determined) indicates the mineral proportions were not calculated due to numerical instability. The blue and white backgrounds indicate the depths at which fluid supply into the mantle wedge was predicted and not predicted, respectively. Srp = serpentine; Tlc = talc; Chl = chlorite; Mgs = magnesite; Dol = dolomite; Qz = quartz.

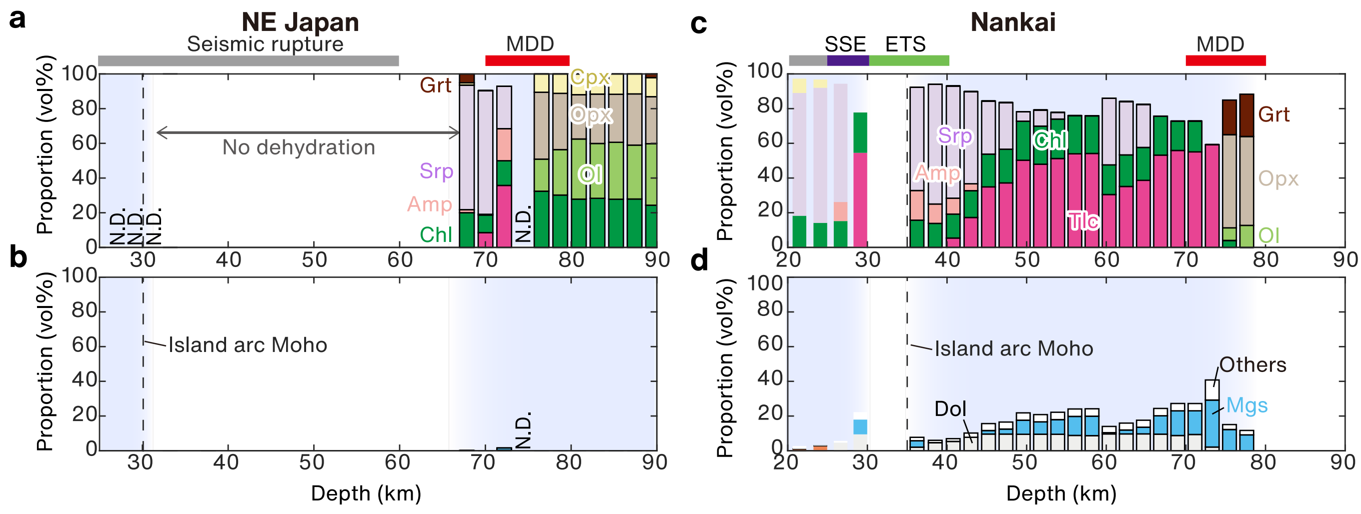


**Supplementary Figure 4. | Model result regarding the infiltration of sediment-derived fluid into anhydrous mantle. a–b** Mineral proportions (**a**) and proportions of carbonate minerals and other minerals (**b**) at the base of the mantle wedge adjacent to subducting sediments in the northeastern Japan subduction zone. **c–d** Mineral proportions (**c**) and proportions of carbonate minerals and other minerals (**d**) at the base of the mantle wedge adjacent to subducting sediments in the Nankai subduction zone. Mineral proportions in regions shallower than the island arc Moho are shown in light colors for reference. MDD = maximum depth of decoupling[1]; MDD = maximum depth of decoupling[1]; SSE = slow slip event[26,27]; ETS = episodic tremor and slip[26,27]. N.D. (not determined) indicates the mineral proportions were not calculated due to numerical instability. The blue and white backgrounds indicate the depths at which fluid supply into the mantle wedge was predicted and not predicted, respectively. Srp = serpentine; Tlc = talc; Chl = chlorite; Mgs = magnesite; Dol = dolomite.

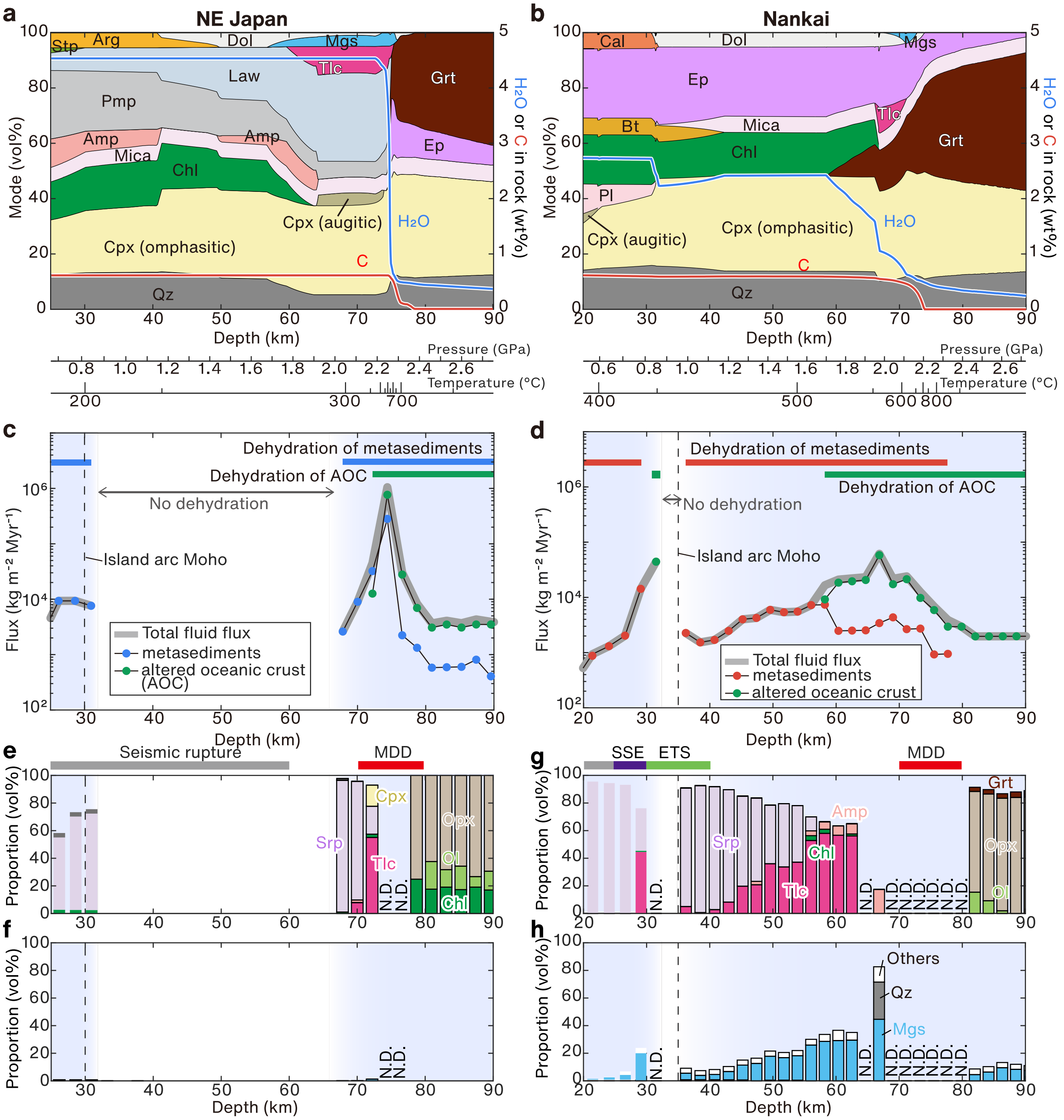


**Supplementary Figure 5.** | **Model results for a subducting slab (metasediments + altered oceanic crust (AOC)). a–b** Mineral phases and $H_2O$ and C concentrations in the AOC for the northeastern Japan (**a**) and Nankai (**a**) subduction zones. **c–d** Fluid flux from the AOC in the northeastern Japan and Nankai subduction zones. The fluid fluxes from metasediments are identical to those in Fig. 2**c**, **d**. The total fluid flux represents the sum of fluid flux from AOC and sediments. In **c**, blue and green bars indicate the depth ranges over which dehydration occurs in the metasediments and AOC, respectively. In **d**, red and green bars indicate the depth ranges over which dehydration occurs in the metasediments and AOC, respectively. **e–f** Mineral proportions (**e**) and proportion of carbonate minerals, quartz, and other minerals (f) at the base of the mantle wedge adjacent to subducting sediments in the northeastern Japan subduction zone. **g–h** Mineral proportions (hydrous minerals (**g**) and carbonate (**h**)) at the base of the mantle wedge adjacent to

subducting sediments in the Nankai subduction zone. In **e–h**, mineral proportions in regions shallower than the island arc Moho are shown in light colors for reference. MDD = maximum depth of decoupling[1]; SSE = slow slip event[26,27]; ETS = episodic tremor and slip[26,27]. N.D. (not determined) indicates the mineral proportions were not calculated due to numerical instability. The blue and white backgrounds indicate the depths at which fluid supply into the mantle wedge was predicted and not predicted, respectively. Qz = quartz, Stp = stilpnomelane, Law = lawsonite, Amp = amphibole, Cpx = clinopyroxene, Chl = chlorite, Ep = epidote, Pl = plagioclase, Bt = biotite, Grt = garnet, Pmp = Pumpellyite, Mgs = magnesite, Gr = graphite, Arg = aragonite, Cal = calcite, Srp = serpentine, Tlc = talc, Chl = chlorite, and Dol = dolomite.

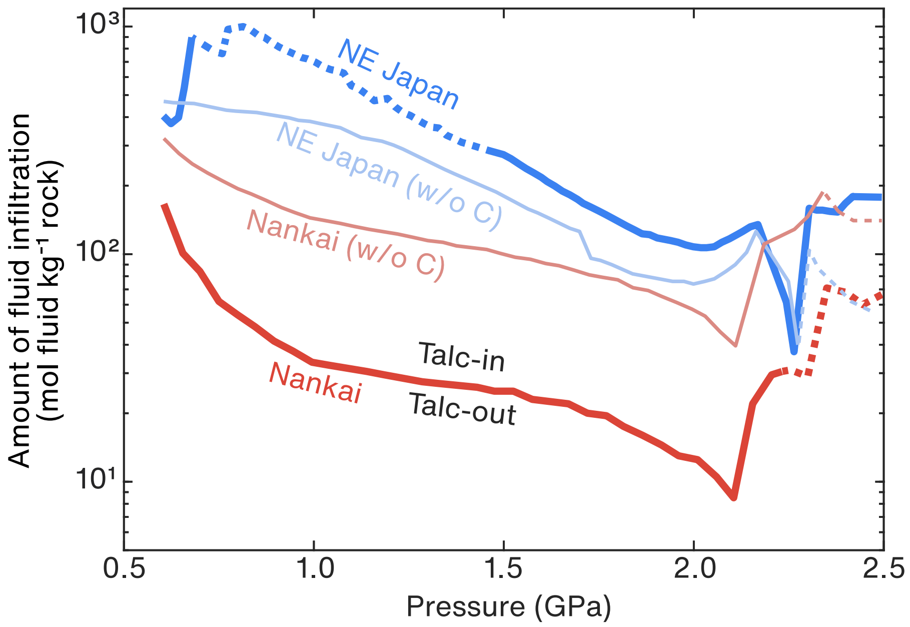


**Supplementary Figure 6.** | **Efficiency of talc formation in the cold and warm subduction zones.** Summary of the fluids required to be equilibrated with the sediments for talc appearance in the mantle wedge. The appearance is defined by the occurrence of 1 vol% talc. Above the line indicates talc occurrence in the rock (talc-in), whereas below the line indicates talc disappearance (talc-out). The light red and light blue lines show calculations that excluded carbon from the calculations. Due to numerical instabilities, the fluid amount for talc appearance could not always be obtained. In this case, the maximum amount of fluid that was not observed during talc formation is shown (dotted line).

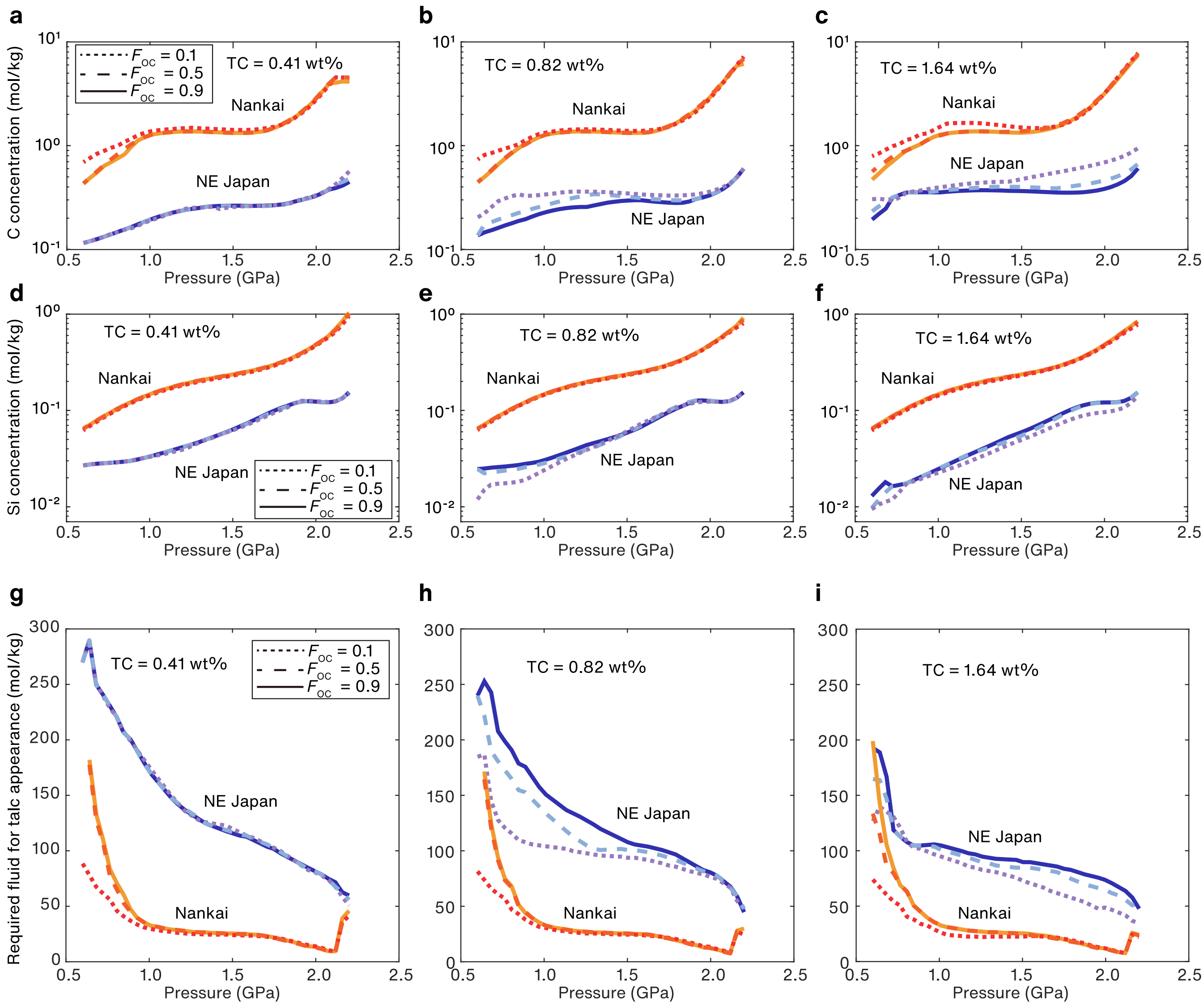


**Supplementary Figure 7.** | **Fluid composition equilibrated with the sediments (GLOSS and a modified GLOSS composition) and the results of infiltration of the fluids into the mantle wedge. a–c** Predicted concentrations of carbon equilibrated with the sediments, with (**a**) 4100 ppm TC, (**b**) 8200 ppm TC, and (**c**) 16,400 ppm TC. **d–e** Concentrations of Si equilibrated with the sediments, with (**d**) 4100 ppm TC, (**e**) 8200 ppm TC, and (**f**) 16,400 ppm TC. **g–i** Summary of 720 calculations of the fluid infiltration from subducted sediments into the mantle wedge, showing the amount of fluid required for talc appearance (mol fluids $kg^{-1}$ rock). The talc appearance is defined by 1 vol% of talc.

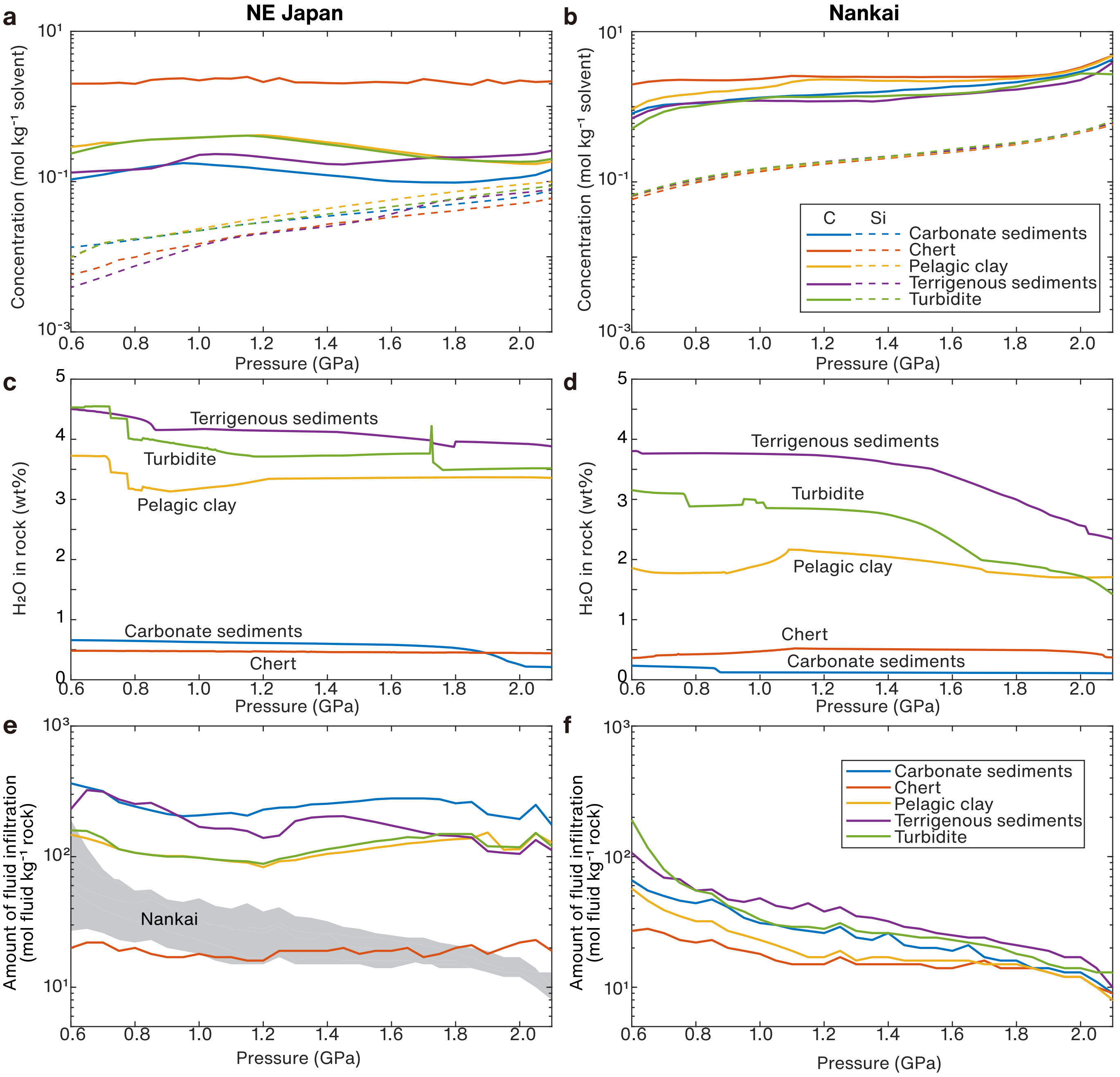


**Supplementary Figure 8. | Composition of fluid in equilibrium with various sediments and the results obtained for fluid infiltration into the mantle wedge. a–b** Concentrations of carbon in fluid in equilibrium with sediments along the slab-top geotherm of the northeastern Japan (**a**) and Nankai (**b**) subduction zones. **c–d** $H_2O$ content in sediments in the northeastern Japan (**c**) and Nankai (**d**) subduction zones. **e–f** Calculated variations of the amount of fluid required for talc formation (mol fluid $kg^{-1}$ rock) in the northeastern Japan (**e**) and Nankai (**f**) subduction zones. The formation of talc is defined by the presence of 1 vol% of talc.

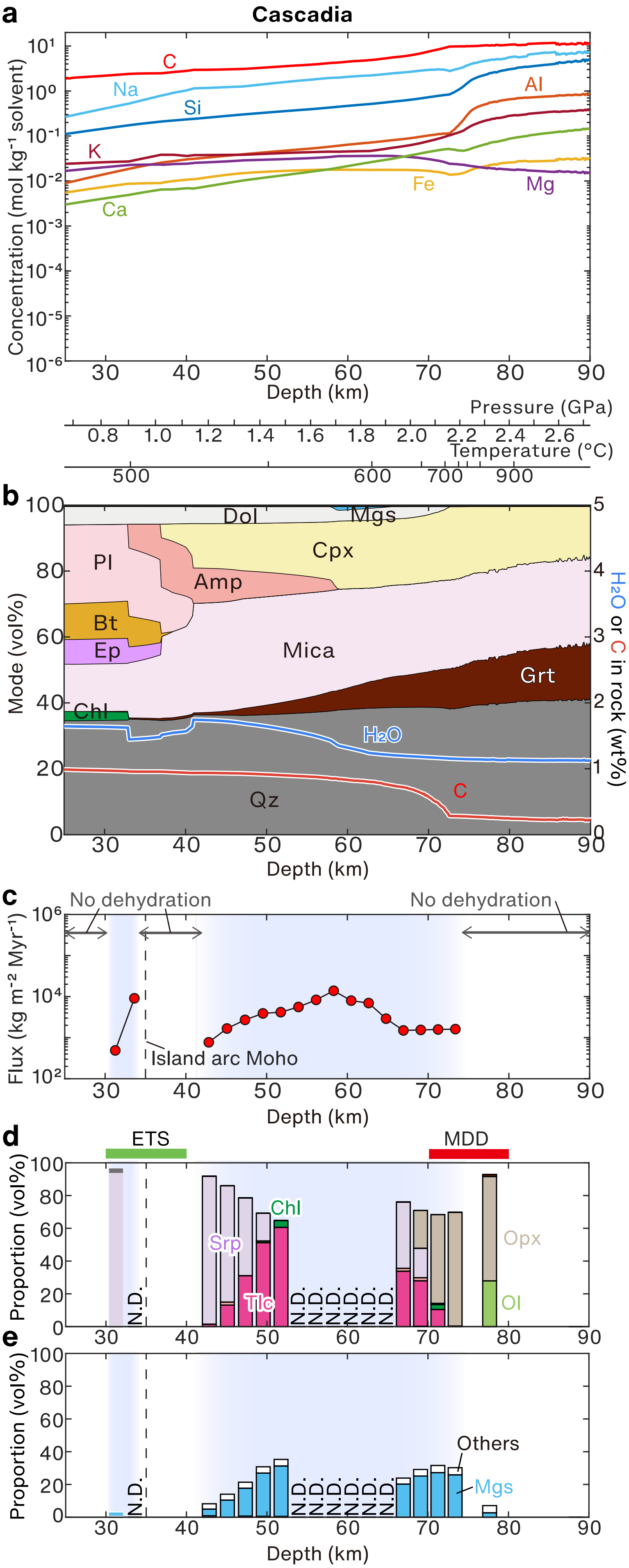


**Supplementary Figure 9.** | **Model predictions for the Cascadia subduction zone. a** Composition of fluid in equilibrium with metasedimentary rocks along the slab-top *P*–*T* path. **b** Mineral phases and $H_2O$ and C contents in the metasedimentary rocks. **c** Flux of fluid derived from subducting sediments. **d–e** Mineral proportions (representative mineral (**d**) and carbonate (**e**)) at the base of the mantle wedge adjacent to subducting sediments. Mineral proportions in regions shallower than the island arc Moho are shown in light colors for reference. MDD = maximum depth of decoupling[1]; SSE = slow slip event[26,27]; ETS = episodic tremor and slip[26,27]. N.D. (not determined) indicates the mineral proportions were not calculated due to numerical instability. In **c–e**, the blue and white backgrounds indicate the depths at which dehydration was predicted and not predicted, respectively. Srp = serpentine; Tlc = talc; Chl = chlorite; Mgs = magnesite; Dol = dolomite.

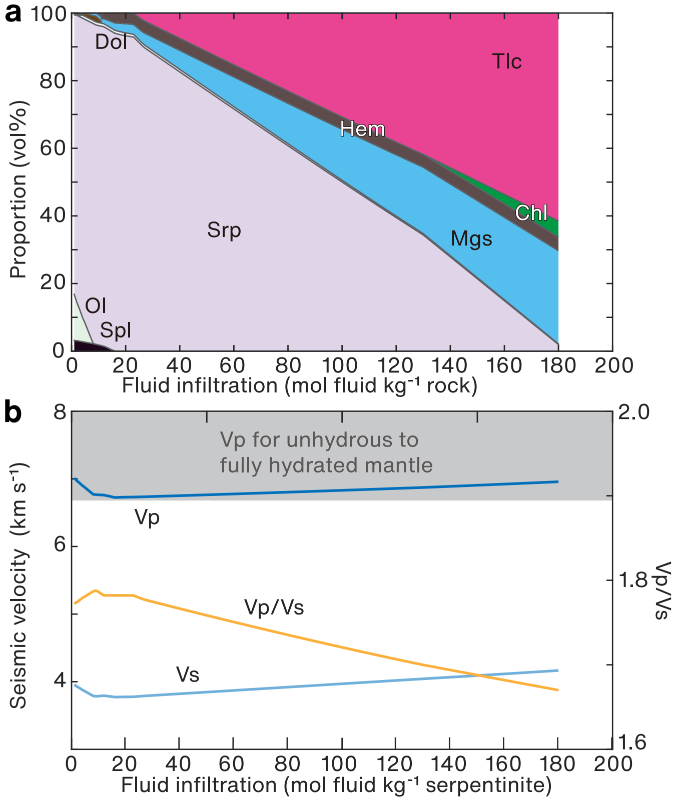


**Supplementary Figure 10.** | **Predicted changes in mineral assemblage and seismic properties in response to the infiltration of serpentinite by fluid derived from subducting sediments. a–b** Thermodynamic calculation of fluid infiltration into a mantle wedge. The figure shows the predicted (**a**) mineral phase changes and (**b**) P- and S-wave velocities when fluid in equilibrium with metasedimentary rocks infiltrates serpentinite of the forearc mantle wedge. The calculations were conducted at 491°C and 1.5 GPa, corresponding to a depth of 50 km in the Nankai subduction zone. The gray region represents the $V_P$ range for anhydrous and fully hydrated (serpentinized) mantle[6].

## Supplementary Tables

### Supplementary Table 1. | Rock compositions used for modeling.

| Lithology<br>References | Nankai sediment [14,18] | NE Japan sediment [14,18] | Mantle wedge harzburgite [28] | Depleted MORB mantle [2,3] | AOC [29,30] | Cascadia sediment [14,19] |
|---|---|---|---|---|---|---|
| $SiO_2$ | 59.72 | 65.43 | 39.82 | 44.90 | 49.23 | 62.78 |
| $Al_2O_3$ | 15.00 | 8.76 | 0.54 | 4.28 | 12.05 | 13.66 |
| $Fe_2O_3$ | 1.34 | 0.97 | 4.44 | 0.17 | 6.99 | 1.39 |
| FeO | 4.03 | 2.93 | 2.89 | 7.05 | 6.05 | 4.18 |
| MnO | 0.59 | 0.39 | - | - | - | 0.06 |
| MgO | 2.22 | 1.87 | 40.16 | 38.22 | 6.22 | 2.53 |
| CaO | 3.92 | 3.23 | 0.29 | 3.50 | 13.03 | 3.80 |
| $Na_2O$ | 1.40 | 2.30 | 0.03 | - | 2.30 | 2.74 |
| $K_2O$ | 3.24 | 1.72 | 0.02 | - | 0.62 | 2.71 |
| $H_2O$ | 6.25 | 10.00 | 11.13 | 0.00 | 4.67 | 5.42 |
| C | 0.42 | 0.33 | 0.00 | 0.00 | 0.00 | 0.31 |
| $CO_2$ | 2.24 | 0.15 | 0.00 | 0.00 | 2.31 | 2.78 |
| Total | 100.37 | 98.08 | 99.32 | 99.00 | 103.47 | 102.38 |
| | | | | | | |
| $Fe^{3+}/Fe_{Total}$ | 0.23 | 0.23 | 0.58 | 0.035 | 0.51 | 0.23 |
| | | | | | | |
| Total carbon ($mg\ kg^{-1}$) | 10280 | 3700 | 0 | 0 | 6300 | 10650 |
| $C^0$ (wt%) | 0.418 | 0.330 | 0.000 | 0.000 | 0.000 | 0.306 |
| $C^{4+}$ (wt%) | 0.610 | 0.040 | 0.000 | 0.000 | 0.609 | 0.759 |
| $F_{OC}$ | 0.41 | 0.89 | 0.00 | 0.00 | 0.00 | 0.29 |
| | | | | | | |
| Input composition used for modeling (mol/kg rock) | | | | | | |
| Si | 9.90407 | 11.10427 | 6.67318 | 7.61033 | 7.91916 | 10.20747 |
| Al | 2.93216 | 1.75271 | 0.10665 | 0.27672 | 2.28437 | 2.61702 |
| Fe | 0.72489 | 0.54016 | 0.96481 | 1.00259 | 1.65985 | 0.73885 |
| Mn | 0.08286 | 0.05606 | - | - | - | 0.00875 |
| Mg | 0.54963 | 0.47207 | 10.03345 | 10.97323 | 1.49164 | 0.61441 |
| Ca | 0.69711 | 0.58750 | 0.05207 | 0.16057 | 2.24551 | 0.66264 |
| Na | 0.45224 | 0.74186 | 0.00968 | - | 0.74217 | 0.86290 |
| K | 0.68499 | 0.37130 | 0.00428 | - | 0.12722 | 0.56136 |
| $H_2$ | 3.45649 | 5.65978 | 6.22101 | 0.00000 | 2.50594 | 2.94082 |
| C | 0.85271 | 0.31409 | 0.00000 | 0.00000 | 0.50692 | 0.86609 |
| $O_2$ | 15.69256 | 16.41926 | 15.53224 | 13.89144 | 14.51986 | 15.68346 |

$F_{OC}$ = OC/(OC + IC) (in mass units).

**Supplementary Table 2. | Summary of solid-solution models used in the thermodynamic model.**

| Mineral | Solution model in the Perple_X | References |
|---|---|---|
| Clinopyroxene (omphasitic) | Omph(GHP) | [31,32] |
| Felspar | feldspar | |
| Epidote | Ep(HP11) | [33] |
| Chlorite | Chl(W) | [34] |
| Mica | Mica(W) | [34] |
| Garnet | Gt(W) | [34] |
| Biotite | Bi(W) | [34] |
| Amphibole | cAmph(G) | [32] |
| Pumpellite | Pu | - |
| Chloritoloid | Ctd(W) | [34] |
| Dolomite | Do(HP) | [35] |
| Magnesite | M(HP) | [35] |
| Olivine | O(HP) | [35] |
| Orthopyroxene | Opx(W) | [34] |
| Spinel | Sp(WPC) | [36] |
| Antigorite | Atg(LE) | [37] |
| Talc | T | Ideal |
| Brucite | B | Ideal |
| Fluid | COH-Fluid | [38] |

**Supplementary Table 3. | Summary of solid-solution models used in the thermodynamic model for altered oceanic crust.**

| Mineral | Solution model in the Perple_X | References |
|---|---|---|
| Clinopyroxene (omphisitic) | Omph(GHP) | [31,32] |
| Clinopyroxene (augitic) | Augite(G) | |
| Felspar | feldspar | |
| Epidote | Ep(HP11) | [33] |
| Chlorite | Chl(W) | [34] |
| Mica | Mica(W) | [34] |
| Garnet | Gt(W) | [34] |
| Biotite | Bi(W) | [34] |
| Amphibole | cAmph(G) | [32] |
| Pumpellite | Pu | - |
| Chloritoloid | Ctd(W) | [34] |
| Dolomite | Do(HP) | [35] |
| Magnesite | M(HP) | [35] |
| Olivine | O(HP) | [35] |
| Orthopyroxene | Opx(W) | [34] |
| Talc | T | Ideal |
| Fluid | COH-Fluid | [38] |

**Supplementary Table 4. | Summary of calculation settings for parameter studies.**

| Calculation No | Geotherm | Sediment composition | Total carbon (wt%) | $F_{OC}$ |
|---|---|---|---|---|
| 1 | Nankai | GLOSS | 0.82 | 0.1 |
| 2 | Nankai | GLOSS | 0.82 | 0.5 |
| 3 | Nankai | GLOSS | 0.82 | 0.9 |
| 4 | NE Japan | GLOSS | 0.82 | 0.1 |
| 5 | NE Japan | GLOSS | 0.82 | 0.5 |
| 6 | NE Japan | GLOSS | 0.82 | 0.9 |
| 7 | Nankai | GLOSS (TC is modified) | 0.41 | 0.1 |
| 8 | Nankai | GLOSS (TC is modified) | 0.41 | 0.5 |
| 9 | Nankai | GLOSS (TC is modified) | 0.41 | 0.9 |
| 10 | NE Japan | GLOSS (TC is modified) | 0.41 | 0.1 |
| 11 | NE Japan | GLOSS (TC is modified) | 0.41 | 0.5 |
| 12 | NE Japan | GLOSS (TC is modified) | 0.41 | 0.9 |
| 13 | Nankai | GLOSS (TC is modified) | 1.64 | 0.1 |
| 14 | Nankai | GLOSS (TC is modified) | 1.64 | 0.5 |
| 15 | Nankai | GLOSS (TC is modified) | 1.64 | 0.9 |
| 16 | NE Japan | GLOSS (TC is modified) | 1.64 | 0.1 |
| 17 | NE Japan | GLOSS (TC is modified) | 1.64 | 0.5 |
| 18 | NE Japan | GLOSS (TC is modified) | 1.64 | 0.9 |

**Supplementary Table 5.** | **Summary of sediment whole-rock compositions used for parameter studies.**

| Lithology | GLOSS (TC was modified) | GLOSS (TC was modified) | GLOSS (TC was modified) | GLOSS | GLOSS | GLOSS | GLOSS (TC was modified) | GLOSS (TC was modified) | GLOSS (TC was modified) |
|---|---|---|---|---|---|---|---|---|---|
| Calc. No | 7,10 | 8,11 | 9,12 | 1,4 | 2,5 | 3,6 | 13,16 | 14,17 | 15,18 |
| $F_{OC}$ | 0.1 | 0.5 | 0.9 | 0.1 | 0.5 | 0.9 | 0.1 | 0.5 | 0.9 |
| Total C (wt%) | 0.41 | 0.41 | 0.41 | 0.82 | 0.82 | 0.82 | 1.64 | 1.64 | 1.64 |
| $SiO_2$ (wt%) | 58.57 | 58.57 | 58.57 | 58.57 | 58.57 | 58.57 | 58.57 | 58.57 | 58.57 |
| $Al_2O_3$ | 11.91 | 11.91 | 11.91 | 11.91 | 11.91 | 11.91 | 11.91 | 11.91 | 11.91 |
| $Fe_2O_3$ | 4.75 | 4.75 | 4.75 | 4.75 | 4.75 | 4.75 | 4.75 | 4.75 | 4.75 |
| FeO | 0.94 | 0.94 | 0.94 | 0.94 | 0.94 | 0.94 | 0.94 | 0.94 | 0.94 |
| MnO | 0.32 | 0.32 | 0.32 | 0.32 | 0.32 | 0.32 | 0.32 | 0.32 | 0.32 |
| MgO | 2.48 | 2.48 | 2.48 | 2.48 | 2.48 | 2.48 | 2.48 | 2.48 | 2.48 |
| CaO | 5.95 | 5.95 | 5.95 | 5.95 | 5.95 | 5.95 | 5.95 | 5.95 | 5.95 |
| $Na_2O$ | 2.43 | 2.43 | 2.43 | 2.43 | 2.43 | 2.43 | 2.43 | 2.43 | 2.43 |
| $K_2O$ | 2.04 | 2.04 | 2.04 | 2.04 | 2.04 | 2.04 | 2.04 | 2.04 | 2.04 |
| $H_2O$ | 7.29 | 7.29 | 7.29 | 7.29 | 7.29 | 7.29 | 7.29 | 7.29 | 7.29 |
| C | 0.04 | 0.37 | 0.21 | 0.08 | 0.74 | 0.41 | 0.16 | 1.48 | 0.82 |
| $CO_2$ | 1.72 | 0.19 | 0.96 | 2.71 | 0.30 | 1.51 | 6.90 | 0.77 | 3.83 |
| Total | 98.44 | 97.24 | 97.84 | 99.47 | 97.72 | 98.59 | 103.74 | 98.92 | 101.33 |
| Input composition used for modeling (mol/kg rock) | | | | | | | | | |
| Si | 9.9031 | 10.0257 | 9.9641 | 9.8010 | 9.9766 | 9.8880 | 9.3977 | 9.8552 | 9.6210 |
| Al | 2.3732 | 2.4026 | 2.3878 | 2.3487 | 2.3908 | 2.3696 | 2.2521 | 2.3617 | 2.3056 |
| Fe | 0.7366 | 0.7457 | 0.7411 | 0.7290 | 0.7421 | 0.7355 | 0.6990 | 0.7330 | 0.7156 |
| Mn | 0.0458 | 0.0464 | 0.0461 | 0.0454 | 0.0462 | 0.0458 | 0.0435 | 0.0456 | 0.0445 |
| Mg | 0.6251 | 0.6329 | 0.6290 | 0.6187 | 0.6298 | 0.6242 | 0.5932 | 0.6221 | 0.6073 |
| Ca | 1.0778 | 1.0911 | 1.0844 | 1.0667 | 1.0858 | 1.0761 | 1.0228 | 1.0726 | 1.0471 |
| Na | 0.7965 | 0.8064 | 0.8014 | 0.7883 | 0.8025 | 0.7953 | 0.7559 | 0.7927 | 0.7739 |
| K | 0.4400 | 0.4454 | 0.4427 | 0.4354 | 0.4432 | 0.4393 | 0.4175 | 0.4379 | 0.4274 |
| $H_2$ | 4.1107 | 4.1616 | 4.1360 | 4.0683 | 4.1412 | 4.1045 | 3.9009 | 4.0908 | 3.9936 |
| C | 0.3471 | 0.3514 | 0.3493 | 0.6871 | 0.6994 | 0.6932 | 1.3177 | 1.3818 | 1.3490 |
| $O_2$ | 15.7488 | 15.6666 | 15.7071 | 15.8971 | 15.6242 | 15.7594 | 15.8833 | 15.5032 | 15.6848 |

**Supplementary Table 6. | Chemical composition and sediment type used in the modeling.** The data were taken from Plank and Langmuir[18], Plank[19], and Clift[14]. See Supplementary Discussion 8 for details.

| Type | Chert | Pelagic clay | Terrigenous sediments | Turbidite | Carbonate sediments |
|---|---|---|---|---|---|
| Site | (Marianas, site 801) | (Marianas, site 801) | (Antilles) | (Alaska, site 178) | (Guatemala, site 495) |
| $SiO_2$ (wt%) | 82.68 | 51.40 | 55.17 | 57.86 | 13.32 |
| $Al_2O_3$ | 3.75 | 15.40 | 20.78 | 15.37 | 0.66 |
| $Fe_2O_3$ | 0.61 | 1.66 | 1.04 | 1.51 | 0.82 |
| FeO | 1.83 | 5.01 | 3.13 | 4.55 | 2.46 |
| MnO | 0.23 | 1.70 | 0.06 | 0.12 | 0.53 |
| MgO | 0.90 | 3.13 | 1.73 | 2.96 | 1.31 |
| CaO | 0.32 | 2.31 | 1.06 | 2.41 | 44.50 |
| $Na_2O$ | 0.85 | 3.64 | 2.58 | 2.84 | 0.44 |
| $K_2O$ | 0.81 | 3.93 | 1.97 | 2.40 | 0.32 |
| $H_2O$ | 8.25 | 9.63 | 7.13 | 9.18 | 1.00 |
| C | 0.05 | 0.05 | 0.10 | 0.40 | 0.00 |
| $CO_2$ | 3.20 | 3.20 | 1.40 | 0.88 | 34.50 |
| Total | 103.48 | 101.07 | 96.15 | 100.49 | 99.86 |
| | | | | | |
| $Fe^{3+}/Fe_{Total}$ | 0.230 | 0.230 | 0.230 | 0.230 | 0.230 |
| | | | | | |
| Total C (ppm) | 9190 | 9190 | 4860 | 6420 | 94091 |
| $C^0$ (wt%) | 0.045 | 0.045 | 0.104 | 0.401 | 0.000 |
| $C^{4+}$ (wt%) | 0.874 | 0.874 | 0.382 | 0.241 | 9.409 |
| $F_{OC}$ | 0.05 | 0.05 | 0.21 | 0.62 | 0.00 |
| | | | | | |
| Input composition used for modeling (mol/kg of rock) | | | | | |
| Si | 13.29878 | 8.46500 | 9.55059 | 9.58392 | 2.22011 |
| Al | 0.71084 | 2.98892 | 4.23939 | 3.00033 | 0.12964 |
| Fe | 0.32010 | 0.89650 | 0.58770 | 0.81857 | 0.44599 |
| Mn | 0.03133 | 0.23712 | 0.00880 | 0.01683 | 0.07482 |
| Mg | 0.21581 | 0.76848 | 0.44648 | 0.73094 | 0.32551 |
| Ca | 0.05514 | 0.40757 | 0.19659 | 0.42767 | 7.94607 |
| Na | 0.26506 | 1.16218 | 0.86588 | 0.91199 | 0.14218 |
| K | 0.16619 | 0.82559 | 0.43501 | 0.50709 | 0.06803 |
| $H_2$ | 4.42548 | 5.28915 | 4.11635 | 5.07111 | 0.55586 |
| C | 0.73940 | 0.75706 | 0.42084 | 0.53193 | 7.84456 |
| $O_2$ | 17.20974 | 15.78229 | 16.08516 | 15.96925 | 14.90340 |

## Supplementary References


1. Wada, I. & Wang, K. Common depth of slab-mantle decoupling: Reconciling diversity and uniformity of subduction zones. *Geochemistry, Geophys. Geosystems* **10**, (2009).

2. Salters, V. J. M. & Stracke, A. Composition of the depleted mantle. *Geochemistry, Geophys. Geosystems* **5**, (2004).

3. Canil, D. *et al.* Ferric iron in peridotites and mantle oxidation states. *Earth Planet. Sci. Lett.* **123**, 205–220 (1994).

4. Lindquist, P. C., Condit, C. B., Hoover, W. F., Hernández-Uribe, D. & Guevara, V. E. Metasomatism and Slow Slip: Talc Production Along the Flat Subduction Plate Interface Beneath Mexico (Guerrero). *Geochemistry, Geophys. Geosystems* **24**, (2023).

5. Condit, C. B., Guevara, V. E., Delph, J. R. & French, M. E. Slab dehydration in warm subduction zones at depths of episodic slip and tremor. *Earth Planet. Sci. Lett.* **552**, 116601 (2020).

6. Abers, G. A., van Keken, P. E. & Hacker, B. R. The cold and relatively dry nature of mantle forearcs in subduction zones. *Nat. Geosci.* **10**, 333–337 (2017).

7. Kelemen, P. B. & Manning, C. E. Reevaluating carbon fluxes in subduction zones, what goes down, mostly comes up. *Proc. Natl. Acad. Sci.* **112**, E3997–E4006 (2015).

8. Gorce, J. S., Caddick, M. J. & Bodnar, R. J. Thermodynamic constraints on carbonate stability and carbon volatility during subduction. *Earth Planet. Sci. Lett.* **519**, 213–222 (2019).

9. Arzilli, F. *et al.* Decarbonation of subducting carbonate-bearing sediments and basalts of altered oceanic crust: Insights into recycling of $CO_2$ through volcanic arcs. *Earth Planet. Sci. Lett.* **602**, 117945 (2023).

10. Müller, R. D. & Dutkiewicz, A. Oceanic crustal carbon cycle drives 26-million-year atmospheric carbon dioxide periodicities. *Sci. Adv.* **4**, 1–8 (2018).

11. Alt, J. C. & Teagle, D. A. H. The uptake of carbon during alteration of ocean crust. *Geochim. Cosmochim. Acta* **63**, 1527–1535 (1999).

12. Jarrard, R. D. Subduction fluxes of water, carbon dioxide, chlorine, and potassium. *Geochemistry, Geophys. Geosystems* **4**, (2003).

13. Kelley, K. A., Plank, T., Ludden, J. & Staudigel, H. Composition of altered oceanic crust at ODP Sites 801 and 1149. *Geochemistry, Geophys. Geosystems* **4**, (2003).

14. Clift, P. D. A revised budget for Cenozoic sedimentary carbon subduction. *Rev. Geophys.* **55**, 97–125 (2017).

15. Clift, P. D. & Hartley, A. J. Slow rates of subduction erosion and coastal underplating along the Andean margin of Chile and Peru. *Geology* **35**, 503–506 (2007).

16. Tewksbury-Christle, C. M., Behr, W. M. & Helper, M. A. Tracking Deep Sediment Underplating in a Fossil Subduction Margin: Implications for Interface Rheology and Mass and Volatile Recycling. *Geochemistry, Geophys. Geosystems* **22**, 1–23 (2021).

17. Agard, P., Yamato, P., Jolivet, L. & Burov, E. Exhumation of oceanic blueschists and eclogites in subduction zones: Timing and mechanisms. *Earth-Science Rev.* **92**, 53–79 (2009).

18. Plank, T. & Langmuir, C. H. The chemical composition of subducting sediment and its consequences for the crust and mantle. *Chem. Geol.* **145**, 325–394 (1998).

19. Plank, T. The Chemical Composition of Subducting Sediments. in *Treatise on Geochemistry* **4**, 607–629 (Elsevier, 2014).

20. Forshaw, J. B. & Pattison, D. R. M. Major-element geochemistry of pelites. *Geology* **51**, 39–43 (2023).

21. van Keken, P. E., Hacker, B. R., Syracuse, E. M. & Abers, G. A. Subduction factory: 4. Depth-dependent flux of $H_2O$ from subducting slabs worldwide. *J. Geophys. Res.* **116**, B01401 (2011).

22. Boyd, O. S. & Clayton, B. S. MinVel—Program to Aggregates, anharmonic P- and S-wave velocity and density for zero-porosity mineral. *U.S. Geological Survey software release* (2019). doi:10.5066/P9FK25WM

23. Abers, G. A. & Hacker, B. R. A MATLAB toolbox and Excel workbook for calculating the densities, seismic wave speeds, and major element composition of minerals and rocks at pressure and temperature. *Geochemistry, Geophys. Geosystems* **17**, 616–624 (2016).

24. Katayama, I., Okazaki, K. & Okamoto, A. Role of mantle carbonation in trench outer-rise region in the global carbon cycle. *Isl. Arc* **32**, (2023).

25. Whitney, D. L. & Evans, B. W. Abbreviations for names of rock-forming minerals. *Am. Mineral.* **95**, 185–187 (2010).

26. Gao, X. & Wang, K. Rheological separation of the megathrust seismogenic zone and episodic tremor and slip. *Nature* **543**, 416–419 (2017).

27. Nishikawa, T., Ide, S. & Nishimura, T. A review on slow earthquakes in the Japan Trench. *Prog. Earth Planet. Sci.* **10**, 1–51 (2023).

28. Deschamps, F., Godard, M., Guillot, S. & Hattori, K. Geochemistry of subduction zone serpentinites: A review. *Lithos* **178**, 96–127 (2013).

29. Hauff, F., Hoernle, K. & Schmidt, A. Sr-Nd-Pb composition of Mesozoic Pacific oceanic crust (Site 1149 and 801, ODP Leg 185): Implications for alteration of ocean crust and the input into the Izu-Bonin-Mariana subduction system. *Geochemistry, Geophys. Geosystems* **4**, (2003).

30. Padrón-Navarta, J. A., López Sánchez-Vizcaíno, V., Menzel, M. D., Gómez-Pugnaire, M. T. & Garrido, C. J. Mantle wedge oxidation from deserpentinization modulated by sediment-derived fluids. *Nat. Geosci.* **16**, 268–275 (2023).

31. Green, E., Holland, T. & Powell, R. An order-disorder model for omphacitic pyroxenes in the system jadeite-diopside-hedenbergite-acmite, with applications to eclogitic rocks. *Am. Mineral.* **92**, 1181–1189 (2007).

32. Green, E. C. R. *et al.* Activity–composition relations for the calculation of partial melting equilibria in metabasic rocks. *J. Metamorph. Geol.* **34**, 845–869 (2016).

33. Holland, T. J. B. & Powell, R. An improved and extended internally consistent thermodynamic dataset for phases of petrological interest, involving a new equation of state for solids. *J. Metamorph. Geol.* **29**, 333–383 (2011).

34. White, R. W., Powell, R., Holland, T. J. B., Johnson, T. E. & Green, E. C. R. New mineral activity-composition relations for thermodynamic calculations in metapelitic systems. *J. Metamorph. Geol.* **32**, 261–286 (2014).

35. Holland, T. J. B. & Powell, R. An internally consistent thermodynamic data set for phases of petrological interest. *J. Metamorph. Geol.* **16**, 309–343 (2004).

36. White, R. W., Powell, R. & Clarke, G. L. The interpretation of reaction textures in Fe-rich metapelitic granulites of the Musgrave Block, Central Australia: Constraints from mineral equilibria calculations in the system. *J. Metamorph. Geol.* **20**, 41–55 (2002).

37. Eberhard, L., Frost, D. J., McCammon, C. A., Dolejš, D. & Connolly, J. A. D. Experimental Constraints on the Ferric Fe Content and Oxygen Fugacity in Subducted Serpentinites. *J. Petrol.* **64**, 1–18 (2023).

38. Connolly, J. A. D. & Galvez, M. E. Electrolytic fluid speciation by Gibbs energy minimization and implications for subduction zone mass transfer. *Earth Planet. Sci. Lett.* **501**, 90–102 (2018).
